# Advances and Challenges in Predictive Modeling for Additive Manufacturing of Dissimilar Metals and Complex Alloys

**DOI:** 10.3390/ma16165680

**Published:** 2023-08-18

**Authors:** Debajyoti Adak, Praveen Sreeramagiri, Somnath Roy, Ganesh Balasubramanian

**Affiliations:** 1 Department of Mechanical Engineering, Indian Institute of Technology, Kharagpur 721302, India; adak.debajyoti@gmail.com (D.A.); somnath.roy@mech.iitkgp.ac.in (S.R.); 2 Department of Mechanical Engineering & Mechanics, Lehigh University, Bethlehem, PA 18015, USA; prs520@alum.lehigh.edu

**Keywords:** additive manufacturing, microstructure simulation, thermal transport, melt pool, process parameter optimization

## Abstract

We present a scrutiny on the state of the art and applicability of predictive methods for additive manufacturing (AM) of metals, alloys, and compositionally complex metallic materials, to provide insights from the computational models for AM process optimization. Our work emphasizes the importance of manufacturing parameters on the thermal profiles evinced during processing, and the fundamental insights offered by the models used to simulate metal AM mechanisms. We discuss the methods and assumptions necessary for an educated tradeoff between the efficacy and accuracy of the computational approaches that incorporate multi-physics required to mimic the associated fluid flow phenomena as well as the resulting microstructures. Finally, the current challenges in the existing approaches are summarized and future scopes identified.

## 1. Introduction

**Figure 1:**
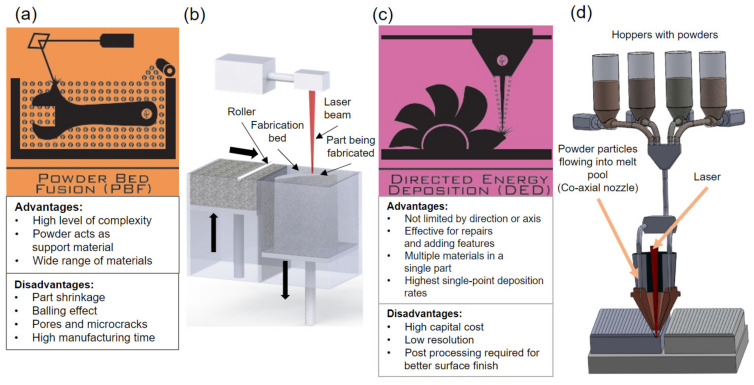
Overview of metal AM processes. (**a**,**b**) Illustration of powder bed fusion process. (**c**,**d**) Illustration of directed energy deposition process [18].

With the emergence of additive manufacturing (AM) as one of the promising platforms to realize the needs for digital manufacturing, the deployment of AM a.k.a. 3D printing has experienced a consistent growth across industries from healthcare to energy to defense, to name a few. In AM, a material is fused layer-by-layer in accord with a computer-aided design (CAD) model to achieve a near net-shaped specimen [1,2,3,4,5,6,7,8,9,10]. Amongst the several benefits over the conventional manufacturing processes (such as casting, forging, machining, etc.), AM eliminates the need for tools or dyes and, in a few cases, assembly to make a component [3,11,12,13,14]. AM is generally classified into several techniques, viz., stereolithography (SLA), powder bed fusion (PBF), direct energy deposition (DED), and material jetting, to list a few. Amongst them, metal AM techniques are particularly intriguing owing to their capabilities in fabricating multicomponent alloy parts; on the other hand, metal AM does need to overcome deep technical challenges associated with the complex solidification cycles and the resultant residual stresses. Nonetheless, products resulting from certain AM processes have demonstrated superior mechanical properties compared to their conventionally processed counterparts [15]. The two most widely employed metal AM approaches include PBF (such as selective laser melting, i.e., SLM) and the directed energy deposition (DED) [15,16]. PBF spreads a thin layer of powder on the substrate followed by a focused heat source (laser or electron beam) that selectively melts and solidifies the powder to create a part. In laser-powder bed fusion (L-PBF) the laser is used as heat source whereas, in the electron beam melting (EBM) process, an electron beam is used as heat source [15,17]. In contrast, the DED mechanism, such as Laser Metal Deposition (LMD), involves focusing the heat source (laser/electron beam/metal arc) to melt the substrate at a precise location and deposit the feed stock (wire/powder) in the melt pool. Salient feature details of these processes and the corresponding (dis)advantages and process variables are illustrated in Figure 1 [18], and typical qualities of the specimens realized by the respective processes are listed in Table 1.

The highly concentrated heat source, along with the rapid melting and solidification in metal AM processes, exert cooling rates ranging from 10^3^ to 10^6^ K/s, resulting in thermal gradients of the order ~10^6^ K/m [1,21]. A high thermal gradient can enable enhanced melting of the alloying elements to produce dense parts with notable mechanical properties [22]. However, with significantly steep thermal gradients, the alloying metals start to vaporize that can generate porosities and cracks in the printed part as the metal vapors are entrapped within, degrading the quality of the component [2,7,12,19,23,24,25]. Reflecting on these challenges, a fundamental understanding of material behavior, typically weldability, during manufacture enables the fabrication of high-quality deposits using metal AM techniques [1]. Also, the need for high-quality feedstock material (powder/wire) devoid of impregnated gases and air pockets poses another challenge for AM. Certified powders for several commercial alloys, viz., nickel-based super alloys, tool steel (H13), stainless steel (316L), and Ti alloys (Ti-6Al-4V), are commercially available; however, acquiring high quality and large volumes of powders for complex and multicomponent alloys, such as high entropy alloys (HEAs), is arduous and expensive. This challenge limits the exploration of the process-design space. Consequently, the process optimization in metal AM becomes time and resource intensive [1,26,27,28,29,30]. Complementary to experiments, computational techniques using the finite element (FE) and finite volume (FV) methods have emerged as viable alternatives to predict the quality of parts and map process parameters to mechanical performance during fabrication [7,31,32,33,34,35,36]. In addition to a comprehensive analysis on the state-of-the-art computational methods applicable to metal AM processes, we address the progress in the AI domain as applicable to the gamut of metal AM techniques. Recent advancements in data science and artificial intelligence promote the application of machine-learning methods (ML) to optimize the AM process [37], assisting in multi-dimensional mapping of process parameters to the product quality. ML algorithms are integrated in three stages of additive manufacturing: (1) geometry design, (2) process parameters optimization, and (3) in situ anomaly detection [38]. 

## 2. Computational Approaches

Traditionally, numerical modeling to predict the temperature distribution, thermal stress and deformation in AM processes has been analogous to multi-pass welding [39]. However, an associated higher degree of complexity in AM arises from the multi-physics phenomena such as the irradiation of the laser beam on the material, heat transfer, melt-pool fluid dynamics, evaporation, and Marangoni effects [40,41,42,43,44,45,46,47], as elaborated below in detail. In this review, we have categorized the computational models into four types. First, three types are based on the scale of modeling. We discuss continuum-scale approaches, which consists of several techniques such as the thermo-physical model, heat-source model, melt-pool model, and structural model, while multi-physics models are constructed by combining these techniques. We present a brief overview of the geometry design and model discretization with different methods (such as FEM and FVM). We note that the optimization of the process parameters is the key objective to realize a smart, efficient, and low-cost AM process. Next, we review microscopic models employed to predict the grain growth and microstructural evolution in the final part. The third category is the multi-scale model where macro-, meso-, and micro-scale models are integrated to predict the overall physics behind the AM process. Finally, the fourth category is based on data driven strategies; recent advances in artificial intelligence (AI) methods enable fast and robust predictions. We discuss machine-learning (ML) process models used in AM. 

### 2.1. Macro Scale Modeling

In continuum-scale modeling, we discuss the modeling of the macro parameters such as stress field, temperature field, and flow field in molten pool. Several models are discussed such as the thermo-physical model, heat-source model, melt pool model, and structural model. Combining these models, multi-physics models are developed. The first step of the modeling is to create the geometry of the model and this geometry needs to be discretized. Different methods such as FEM and FVM are widely used to simulate these models. The aim of these simulations is to find a relation between the process parameters with the quality of the final printed part. Optimization of the process parameters is the major challenge in the AM process to have an accurate product with no defects.

#### 2.1.1. Part Geometry, Discretization, and Boundary and Initial Condition

**Figure 2:**
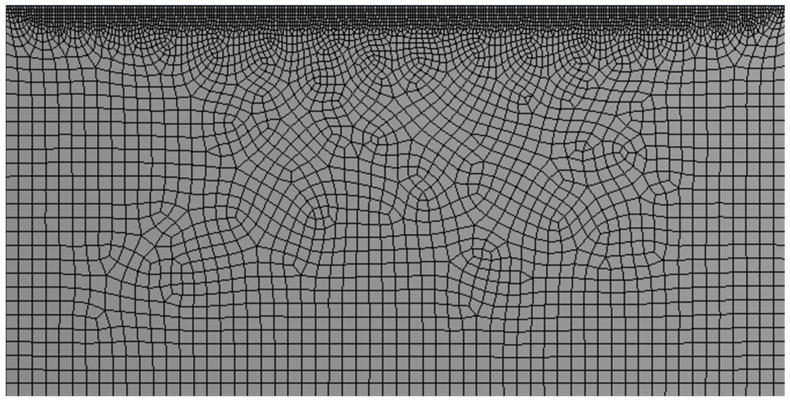
A mathematical equivalent of a geometric (CAD) model with bimodal discretization representing coarse and fine elements for a face of a cuboid.

A numerical model initiates with a discretized (viz., elements) mathematical equivalent of a geometric (CAD) model called “*mesh*”. First, a desired geometry is created with CAD software (such as DesignModeler, spaceclaim, SOLIDWORKS, PTC Creo, CATIA, AutoCAD, etc.). Then the geometry is discretized by creating proper mesh. The elements embodying the mesh assume several geometric shapes, such as, a triangle or a rectangle in two dimensions, with tetrahedrons, hexahedrons etc., subdomains in 3D. However, rectangles and their sub-domain hexahedrons are preferred wherever possible to accurately represent the solution with an enhanced computational efficiency. Stresses enacting on these elements are evaluated from the forces acting on the nodes, which are integrated over the entire geometry to predict the dynamics of a part under specified loading conditions. It is important to incorporate a finer mesh to increase the accuracy of calculations, albeit at a substantial computational cost. The choice of coarse/fine elements typically depends on the diverse features of the part geometry, as illustrated in Figure 2. The finer regions in the figure represent the powder particles that are spread across the substrate of coarser elements. In other words, the mesh employed to discretize the part depends on the physical properties such as the shape, but also on the underlying physics being replicated. For instance, when moving a heat source over the surface of an object, we do not implement a fine mesh throughout the geometry, but only for the path of the heat source with the rest of the geometry represented by coarser mesh. Employing finer elements for powder particles alongside coarser elements for the substrate improves the computational efficiency and permits capturing the accurate temperature fields in the laser irradiated zones (points of interest). Therefore, an optimization scheme to include a collective (non-uniform/adaptive) of coarse (large elements) and fine (small elements) mesh during discretization is crucial for computationally efficient and accurate predictive methods [7,22,48,49,50,51]. For instance, an adaptive mesh refinement (AMR) scheme leverages finer mesh in stress concentration zones, with the residual areas discretized into coarser elements crafting a computationally efficient model. Extending further, AMR can be classified into two techniques, viz., static and dynamic AMR, whence a dynamic AMR adapts to changing part geometries in real (simulation) time [51,52]. A comparison of the computing times between fine and coarse mesh schemes reveals that a coarse adaptive mesh is notably efficient with predictions resembling the ground truth (details listed in Table 2). Hajializadeh and Ince, 2018 [51], simulated an 18-layer L-shape part by using adaptive mesh coarsening and compared the computation time with a conventional fine uniform mesh model in a FEM-based DMD process. An ideal implementation of these techniques in AM simulations requires an AMR scheme with the finer mesh dynamically adapting to the shape of the melt pool, and subsequently moving with the heat source [53]. Besides melt pool, it is important to refine the areas with high thermal gradients and stresses, generally near the heat sinks.

To simulate the model accurately, the most important thing is to set the boundary and initial conditions properly. Boundary conditions specify the environmental constraints and their associated heat transfer mechanisms when modeling an AM process [54]. While conduction through substrate and the previously solidified layers is the dominant heat-transfer mechanism for any AM process, dissipation of a fraction of the total energy through convection and radiation to the surrounding environment is inevitable [7,35]. 

The governing equation of heat transfer for isotropic solid material with temperature independent properties is given in Equation (1) [7].

(1) $$\frac{\partial T}{\partial t}=\frac{k}{c\rho }\left(\frac{\partial^{2}T}{\partial x^{2}}+\frac{\partial^{2}T}{\partial y^{2}}+\frac{\partial^{2}T}{\partial z^{2}}\right)+\frac{1}{c\rho }\frac{\partial Q_{v}}{\partial t}$$

where, *k* is the thermal conductivity, *c* is specific heat, *ρ* is density, and *Q_v_* is consumed thermal energy per unit volume (due to the heat source).

A simple thermal transport model incorporating all modes of heat transfer for an AM process models the laser-irradiated surface being subjected to a condition presented in Equation (2), and assumes no heat transfer to the bottom surface [44,55,56].

(2) $$-k\frac{\partial T}{\partial n}=h_{c}\left(T-T_{0}\right)+\varepsilon_{\theta }\sigma \left(T^{4}-T_{0}^{4}\right)$$

where, *k* is the thermal conductivity, $\frac{\partial T}{\partial n}$ is the temperature gradient, *h_c_* is the convection coefficient, *T*_0_ is the room temperature, $\varepsilon_{\theta }$ is the emissivity, and *σ* is the Stefan-Boltzmann constant. Typically, to limit modeling complexity, convection and surface radiation are ignored with only minimal loss in the predictive accuracy for modeling of melt pool.

From the literature, it is observed that thermo-mechanical simulation and inherent strain method-based (discussed later in the structural model section) simulation are mainly carried out using the finite element method (FEM) model. This thermo-mechanical FEM model is used to couple the thermal and mechanical conditions of the AM process to enable an accurate prediction of thermal stress and deformation induced in the printed part [35,57,58]. On the other hand, thermo-fluid dynamics phenomena involved in AM is generally modeled using the finite volume method (FVM) model [59,60,61]. This model incorporates many thermo-fluid characteristics such as conductivity, melt-pool convection flow, wettability, thermo-capillary forces, etc., which makes it much more complicated, and subsequently computer intensive. Moreover, micro-scale models have gained attention due to their ability to accurately predict the local deformation and instabilities within the printed part. For microstructure simulations the commonly used approaches are Lattice Boltzmann-cellular automata and Phase field (PF) models, and more recently using a hybrid FEM/FVM model [62,63,64,65,66,67].

#### 2.1.2. Process Parameters

**Figure 3:**
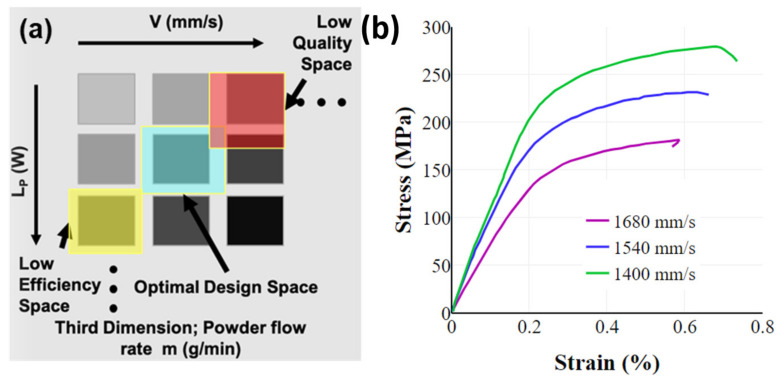
(**a**) Design of experiment matrix [18]. (**b**) Increasing the speeds beyond the optimal design speed results in incomplete melting of powders, thus reducing the density and consequently being detrimental to the mechanical properties of the fabricated component [71].

AM machine variables such as the power of the heat source, scan speed, hatch distance, and powder flow rate, to list a few, are crucial to obtaining crack- and defect-free dense deposits [7,44,68]. Table 3 catalogues the crucial parameters that are required to be optimized for a PBF process. Optimal selection of the parameters to construct a process workspace for producing a certifiable specimen requires design of experiments (DoE), as illustrated in Figure 3a [18], with the process variables in the design matrix. Design of experiment matrix demonstrates that a high scan speed coupled with low power results in a low-quality deposit typically with incomplete melting of the material; a low speed with a high power produces a low-efficiency space contributing to over heating or melting the material. Thus, identifying an optimal design space (process parameter window) is vital to achieve high-density parts [18]. While DoE may mandate several iterations [15,34,44,69,70], the availability of multiple metal AM processes, advocates normalizing these parameters to achieve material-specific properties, eliminating machine-to-machine variability. Such standardizing results in global variables, *viz.*, energy deposition density (*E*) and powder deposition density (*P*) derived from laser power (*L_p_*), scan speed (*V*), layer thickness (*t_L_*), and beam (*d_b_*) and nozzle (*d_n_*) diameters [1,18]. The correlations provided in Gorsse et al. [1] are listed as Equations (3) and (4). Here, m is powder flow rate (g/min), which is the process-specific parameter describing the powder supplied to the melt pool per unit time and applicable only for DED processes. While these normalized variables could be used as a material-specific property when porting between processes, we note that only *E* could be adopted from DED to PBF; an additional variable *P* should be determined when translating from PBF to DED. While the expression of *E* in PBF is akin to DED, the beam diameter (*d_b_*) is to be replaced by hatch spacing (*h*). Cross-utilization of these parameters mandate additional DOEs, and can only offer predictive estimates and not a direct correlation due to machine-specific constraints.

(3) $$P=\frac{m}{V\times d_{b}\times t_{L}}{\left(\frac{d_{b}}{d_{n}}\right)}^{2}\left(\frac{gm}{{mm}^{3}}\right)\;(\mathrm{for}\mathrm{DED})$$

(4) $$E=\frac{L_{P}}{V\times d_{b}\times t_{L}}\left(\frac{J}{{mm}^{3}}\right)\;\left(\mathrm{for}\mathrm{DED}\right);E=\frac{L_{P}}{V\times h\times t_{L}}\left(\frac{J}{{\mathrm{mm}}^{3}}\right)\;(\mathrm{for}\mathrm{PBF})$$

Besides process parameters, scanning strategies influence the induced thermal stress and temperature gradient (scanning pattern, scanning direction, and scanning vector length) [15,36,72,73,74,75]. In addition, lower scan speeds lead to longer interaction times for the laser with the metal and consequently increase the density of the material improving the mechanical properties (Figure 3b [71]). However, with much lower scanning speeds, the alloying elements start to vaporize resulting in the formation of keyholes and porosities in the printed part [76]. The distribution of absorbed thermal energy varies based on the powder bed’s relative density and reflectivity in the successive powder layers [77]. The processing challenge intensifies due to the metal powders’ high thermal conductivity, surface tension, and laser reflectivity [78]. Additionally, the SLM process for AlSi_10_Mg powder is particularly challenging to control, given its high reflectivity and thermal conductivity. This complexity stands in contrast to the production of other metal powders such as stainless steels or titanium alloys [79]. While SLM has proven its versatility across various materials, encompassing metals, polymers, and ceramics, the intricate processing of materials featuring elevated thermal conductivities and high melting points, such as pure copper, faces notable challenges. Besides the rapid heat dissipation problems, the reflectivity of copper to conventional laser light near infrared is very high, resulting in low deposition of laser energy in the materials during melting. In order to impart higher laser energy density for manufacturing dense copper parts, an enhanced laser output power with reduced scanning speed, layer thickness, and hatch spacing are needed [80]. 

The absorption efficiency and surface emissivity govern the energy entering and exiting the AM platform [27]. Since the heat source acts on the powder layers in PBF systems, both the effective heat conduction inside the layer as well as the laser absorption should be considered in the models [81]. During AM, the laser beam’s absorption by the workpiece is influenced by factors such as the powder-particle size distribution, feed rate, laser beam wavelength, and power density distribution. Given the significant number of variables that govern the interactions between the laser beam and the workpiece, constructing an exhaustive model that encompasses all possible powder feeding scenarios in AM is limited in accurately replicating the intricate physical processes at play. Therefore, a specific model is employed to address this problem [82]. The choice of process parameters also has a marked impact on the intermediate dimensions, such as layer height and width of the build, that is nontrivial for complicated geometries.

Porosities form one of the predominant challenges in metal AM, specifically the PBF; as powder is distributed on the bed prior to melting the substrate, the substrate is irradiated to the laser through the powder particles, creating a few pores during solidification and resulting in an utmost ~99.8% dense components post process-parameter optimization [83]. This outcome is due to the inherent technology limitation, where the powder is laid on the build plate and the laser must pass through the powder into the previous layers to deposit material. With the high scan speeds employed, part densification persists to be a challenge. In addition, spherical powder particles used in the process to enhance flowability, the packing factor and the powder morphology contribute significantly towards densification of the printed component. Such defects compromise the build rate by employing lower layer heights to achieve optimal mechanical properties [83]. In contrast, DED directs a focused heat source and subsequently deposits the feedstock, producing parts with relatively higher densities and build rates. On occasion, it may be possible to achieve densities >99.8% in PBF, but only in highly ideal conditions and may not be reproducible in terms of structural/functional properties/features. Besides porosities, a lack of fusion is another major defect found in AM parts [84]. Such defects occur mainly due to three reasons. First, the use of very high energy deposition in keyhole-mode melting in the AM process. These keyholes might form and collapse repeatedly and result in the formation of porosities in the deposited layer [85]. Second, during powder atomization process, gases might become entrapped within the powder particles, which may lead to a porous and defective final part [86]. Third, if the melt pool fails to penetrate across the layers deposited on the substrate, a lack of fusion can be seen in the printed part [86,87]. Mukherjee et al. [88] propose a relation between the melt-pool geometry and the lack of fusion. The “lack of fusion index” (LF) is given by the Equation (5).

LF = Melt pool depth/Layer thickness(5)

For a higher LF value, the lack of fusion voids decreases, and the LF value can be increased by incorporating a larger melt pool, which results in the proper bonding between the successive deposited layers [88]. 

The other defects observed in the selective laser sintering (SLS) AM process are balling (lump formation of powder), tearing (propagation of crack due to thermal stress), rough surfaces, and poor cohesion, which leads to the printing of faulty components. The effect on the part properties include shrinkage, porosity, and dimensional inaccuracy [66,69,89,90,91,92].

From the experiments on SLM with SS316L, Miranda et al. [29] observed that presence of porosity is higher for lower laser power. Increasing the power to 90 W, reduced the porosity and increased the density and mechanical properties. However, for higher scanning speed the part became more defective with lower hardness and lower shear strength [29,93]. Moreover, high scan spacing leads to lower shear strength and lower density. For large scan spacing, adjacent lines of powder do not bond well with each other. Scan spacing also affects the microstructural growth, with coarser structures observed for higher scan spacing [29].

Likewise, inaccurate process parameters during fabrication can lead to several defects such as lack of fusion, key holes, balling, etc. For instance, an inadequate laser power (<80 W) during SS316 printing contributes to improper fusion effecting in ~50% reduction in strength [71,94]. On the other hand, employing high laser power in complicated geometries containing overhangs can promote warping of material due to the instabilities in the melt pool [95]. Thus, the optimization of process parameters is extremely important to achieve high-quality deposits. Surface finish for AM fabricated parts is another concern due to the layered fabrication strategy. A transient temperature field simulation in PBF of copper powder suggests a better surface finish can be achieved with high scanning speeds with multi-layer sintering [96].

#### 2.1.3. Thermophysical Models

Variation of thermophysical properties like thermal conductivity, latent heat, and specific heat capacity are important to consider during processing as they associate density, microstructural features, and the resultant material properties (e.g., porosity and residual stress) on the fabricated component [2,6,7,22,34,44,49,58,79,97,98,99,100,101,102,103,104,105,106]. The rapid heating and cooling in AM lead to disparate melting and solidification cycles resulting in accelerated phase changes (powder to liquid to solid), which affect the porosity and density in the specimen. Therefore, understanding the evolution of density during printing can shed light on the thermal conductivity and laser absorptivity (of powder material) as a function of density or porosity [50,73,107,108,109].

Conduction through bulk material is the primary mode of heat transfer in AM and variations in thermal conductivity as a function of density influence the microstructures that are produced [73,109,110]. The effective thermal conductivity of a material as a function of conductivity of the solid material *k_s_* (*T*) and porosity *β* is presented in Equation (6) [50].

(6) $$k_{eff}=k_{s}\left(T\right)\times \left(1-0.2\beta -1.73\beta^{2}\right)$$

Akin to porosities in a bulk material, the extremely small contact areas in the feedstock material limit the thermal conductivity in the powder and consequently retard the cooling rates [49,73]. Also, particle size and powder packing influence the heat conduction in PBF [111], with the conductivity in the solid particles. Incorporating the amount of energy released or gained during phase change (viz., latent heat) can further improve the model and provide a quantitative understanding on the variation of thermal conductivity and specific heat capacity. These approaches can assist in mapping process parameters to the structural properties. 

Enthalpy methods model the liquid-to-solid phase change by tracking the enthalpy of the system instead of temperature, thus enabling the calculation of latent heat during the phase change. Implementation of this model in FEM is relatively straightforward by employing the enthalpy equations instead of heat-transfer equations [81,112]. These methods facilitate, also known as the enthalpy-porosity technique, the modeling of solid–liquid mushy zones by considering the mushy zone as a porous medium with the liquid volume fraction considered as the porosity of the porous medium [113,114]. In addition, an equivalent specific heat can be introduced by considering the effect of latent heat on temperature field, enhancing the accuracy of the model, however at an increased computational expense [115].

#### 2.1.4. Heat Source Model

**Figure 4:**
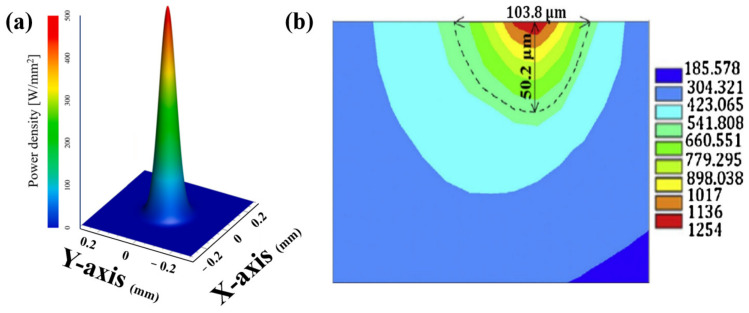
(**a**) A Gaussian model: the intensity of laser beam is at its highest in the center (0,0) of the focal point, while retarding along the radial direction [89]. (**b**) The temperature profile varies according to the intensity of laser (Gaussian function) [116].

Physically, the intensity distribution of the laser on the material conforms to a Gaussian model (as reproduced in Figure 4a [89]), with the highest intensity of the laser and consequently the peak temperature recorded at the center of the focal point (Figure 4b [116]), exponentially reducing in the radial direction [6,117,118]. The heat flux can be expressed as a function of space and time (Equation (7)) to construct a resulting molten-pool (Goldak) model [103,119,120].

(7) $$q\left(r\right)=q_{max}e^{-kr^{2}}\left(\frac{J}{mm^{2}s}\right)$$

Here, *q_max_* is the maximum heat flux (J/mm^2^∙s), *k* is concentration factor (1/mm^2^), with *r* being the distance between a point and center of the heat source (mm). The geometry of the melt pool is highly dependent on the scan speed and its length increases with the increasing scanning speed, with a notable decrease in depth and width [49]. Extending this method, Irwin and Michaleris [121] introduced a line-based heat input model to accurately predict the heat distribution in the PBF process without compromising computational efficacy.

For a volumetric moving heat source in the powder bed, Goldak’s double ellipsoidal heat source model can be used, given in the Equation (8).

(8) $$q\left(x,y,z\right)=\frac{6\sqrt{3}Q}{abc\pi \sqrt{\pi }}e^{-3\left(\frac{{\left(x-v_{x}t\right)}^{2}}{a^{2}}+\frac{{\left(y-v_{y}t\right)}^{2}}{b^{2}}+\frac{z^{2}}{c^{2}}\right)}$$

where, *a*, *b*, and *c* represent the semi-axis of the ellipsoidal heat source along *x*, *y*, and *z* directions, respectively, in the powder bed.

#### 2.1.5. Melt-Pool Models

**Figure 5:**
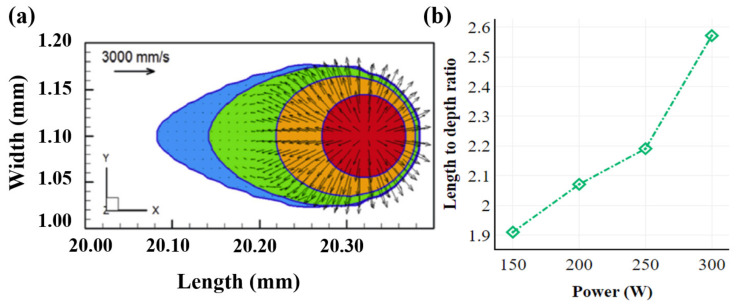
(**a**) Illustration of the comet tail profile of the melt pool [89]. (**b**) Relation between power and length (*l* mm)-to-depth (*d* mm) ratio suggests that *l/d* increases with increasing power offering recommendations for the layer height to be employed during deposition [79].

The melt pool is the region of the alloy where a phase change to liquid state occurs as a consequence of irradiation by the heat source, often with a comet tail profile (displayed in Figure 5a) [34,89,122]. The different heat sources (laser/electron beam) often result in a diverse beam. For instance, using an electron beam as a heat source produces a melt pool with diameters ~10% larger than that of laser processes [102]. The length-to-depth ratio of the melt pool increases with increasing power (Figure 5b [79]). However, the directional and concentrated nature of any heat source limits the melt pools to <5 mm in diameter. This results in rapid heating and cooling cycles lasting a few milliseconds, and are controlled by the process parameters, specifically the power and the scan speed employed. For example, a higher scan speed can result in a longer tail in the melt pool, with the depth and width reduced [49], and vice-versa [56,123]. The areas preceding the melt pool realize a higher temperature gradient relative to the areas following the melt pool; hence, understanding the fundamentals of melt-pool formation and the associated dynamics elucidate the grain-growth mechanisms and the properties of the fabricated parts [61,98,124]. 

The size and the compactness of the powder bed influence the thermal conductivity through the substrate underneath, which also triggers the melt-pool characteristics. An ideal model to predict the melt-pool features should consider the fluid dynamics effects including Marangoni and buoyancy [125]. The Marangoni effect [82,126,127] is the mass flow mechanism due to surface tension gradient caused by the temperature gradient on the surface. The Marangoni effect becomes prominent as the melt-pool size and depth increase because of high-energy deposition, leading to a higher tangential velocity at the top surface. The mathematical model (Equation (9)) for the Marangoni effect includes the surface shear stress of the melt pool [127].

(9) $$\tau_{r}=-\mu \left(\frac{{\partial u}_{r}}{\partial z}\right)=\left(\frac{\partial \gamma }{\partial T}\right)\left(\frac{\partial T}{\partial r}\right)$$

Here, *τ_r_* is the surface tension (N/m^2^), $\left(\frac{\partial \gamma }{\partial T}\right)$ is the surface tension gradient (N/m-K), and $\left(\frac{\partial T}{\partial r}\right)$ is the temperature gradient along the melt pool surface (K/m). Collectively, these effects exert rapid cooling and heating cycles generating a compressive stress within the melt pool when cooling, followed by a tensile stress near the solid–liquid interface to balance the force and momentum. Considering the Marangoni effect yields a relation between the energy density of the laser and the melt-pool depth and size, aiding in optimizing the laser parameters [127].

The study of melt pool includes a large number of thermo-fluidic phenomena such as fluid flow, radiation, vaporization, and variable material properties [34,128], which should be included in the finite volume model. Commercial software available for such simulations include Flow-3D, Fluent, etc. [89]. The powder-scale model incorporates the multi-physics phenomena using the ALE3D, Open-FOAM, and the LBM (Lattice Boltzmann method), and it also incorporate thermo-fluid code with mass-momentum energy in transient form [129]. Temperature-dependent properties have been simulated for Ti-6Al-4V, IN718, and AlSi_10_Mg. Körner et al. [130] used in-house code by using the Lattice Boltzmann method (LBM) to study the effect of beam power, beam velocity, and layer thickness on the wall formation. Likewise, in Los Alamos National Laboratory, Truchas code was developed to solve the point heat-source scan strategy for IN718 in PBF-EB [74,131]. They have modeled solidification with a non-isothermal phase change in the mushy zone and simulated for various process parameters to study the variation of temperature gradients. Ahmadi et al. [71] studied the properties of SS316L printed using the SLM process using the cohesive zone model (CZM) to predict the interaction between the pool boundaries. It was observed that defects generally start to occur in the pool boundaries as they are much weaker than the grain boundaries. These predictions agree well with the experimental data, but the computational cost is the major challenge for scaling up these models. Hence, more efficient models are required that can continue to improve the accuracy of the simulations within reasonable computational demands. Other FEM-based commercial software that are often employed to replicate such physical phenomena include ANSYS, MSC Marc, COMSOL Multiphysics, and Abaqus [89]. 

#### 2.1.6. Structural Model

Process parameters employed during metal AM drive the thermal profiles and subsequently induce residual stresses, warping, inaccuracies in geometric shape, etc. [24]. Residual stress generated inside an AM printed part mainly arises from three underlying mechanisms. First is the spatial temperature gradient generated from the repeated heating and cooling by the moving heat source. Second, thermal expansion and contraction due to rapid cooling and heating; and third is the nonuniform distribution of inelastic strains, force equilibrium, and stress–strain constitutive behavior [84]. However, the residual stress can be minimized by pre-heating the base plate [132]. Residual stress may cause bending in the printed part, which can be avoided by using a thicker base plate [133]. The residual stress is also responsible for delamination and cracking in high-stress areas. Part geometry, energy deposition methods, material properties, and process parameters are responsible for generation of residual stresses in the printed part [44,57,89,99]. In general, the residual stress is recorded to be very high at the edge where the printed part joins the base plate. When the residual stress exceeds the yield strength, delamination and cracking may occur in the build [89]. The accuracy of the model can be enhanced by incorporating the effects of residual stress relaxation. 

Yakout et al. [134,135] investigated the influence of the thermal properties on residual stress of Invar 36 and SS 316L produced using the SLM process. They have examined the microscopic residual stress generated inside the printed part using the X-ray diffraction (XRD) method. Using this method, stress tensor is measured based on the lattice strain measurement. Gusarov et al. [136] reported that residual stress is higher in the scanning direction as compared to the transverse scan direction. Nowadays, inherent strain (IS) methods are widely used to simulate the thermal stress [137,138]. This method, which was created for a welding simulation, is now modified to be adopted to the PBF process simulation. Here, the thermal stress is simulated to the component scale with inherent strain (residual plastic strain) tensor, which activates in the discrete hatching region of the mechanical model layer-by-layer. Keller et al. [137] coupled this IS method to a multiscale model. From the results, the value of residual distortion was very close to the experimental measurement. 

Table 4 lists some of these properties and their effects on the final part, which result from shrinkage and stress-induced deflection and can be controlled by optimizing the process parameters [139,140]. Predicting these effects employing quasi-static elastoplastic models prior to fabrication facilitates a reduction in the above-mentioned artifacts to achieve a high-quality part [2,28,141]. Quasi-static elastoplastic models in general constitute a twofold process and can be categorized into (a) coupled and (b) weakly/uncoupled methods [35,90,103,117]. A coupled analysis considers the effects of thermal expansion on the mechanical properties within the model, while the weakly coupled model assumes them to be independent and requires the user to serve as a middleman. This approach showcases the weakly coupled method as an inexpensive and preferential option [99,142]. Equation (10) through (13) represent the governing mechanisms for deriving the stress tensor from the thermal profiles [115].

(10) ∇σ=0

where *σ* refers to a second-order stress tensor and is calculated from the thermal strains and the elastoplastic behavior. (Equation (11)) with *C* and *ε^e^* being the 4th order material stiffness tensor and the second-order elastic stain tensor, respectively.

(11) $$\sigma =C\varepsilon^{e}$$

Further, the total strain tensor *ε* (Equation (12)) considers the elastic strain *ε^e^*, the plastic strain *ε^p^*, and the thermal strain *ε^th^* [27] with the thermal strain as displayed in Equation (13) with *T* and *T*_0_ being the current (at time *t*) and the initial temperatures, respectively. Few complexities in these models include estimating the final distortions without the base plate and the supports [49].

(12) $$\varepsilon =\varepsilon^{e}+\varepsilon^{p}+\varepsilon^{th}$$

(13) $$\varepsilon^{th}=\alpha \left(T-T_{0}\right)$$

These are the basic driving equations for deriving more complex equations. While most of the studies are limited to single layer-single track depositions, these methods have proven important to understand the associated complexities revealing the samples processed using the EBM technique assume a relatively low residual stresses than the SLM as a consequence of varying cooling rates [7]. However, many commercial software (e.g., Simufact, Amphyon, GeonX etc.) are now available for the residual stress distortion simulation [57]. 

#### 2.1.7. Multi-Physics Modeling

Efficiency and accuracy of metal AM processes can be maximized by optimizing the process parameters. Predicting the defect formation and instabilities are the major research thrusts to optimize the process parameters [31,47]. The different combinations of process parameters are integrated with micro-scale models to predict the in situ defects as well as large-scale anomalies and instabilities (such as porosity, balling, and spatter) generated in the final product [42,57,130]. The AM process consists of thermo-fluidic phenomena such as irradiation of the laser beam on the material, plasma plume recoil pressure, heat transfer, metal phase change, melt-pool fluid dynamics, evaporation, wettability, Marangoni effect, thermo-capillary forces, etc. [31,47,57]. Simulating, calibrating, and optimizing the model consisting of all these multi-physics phenomena with different sets of process parameters requires significant computational power. 

Thermal and mechanical features in the AM process can be coupled using a thermo-mechanical FEM model. Residual stress and deformation can be determined accurately by employing accurate thermo-mechanical properties in the model. Furthermore, viscous dissipation phenomena can be considered through this coupled model [57]. Hussein et al. [49] simulated a FEM model for the successive deposited layers to study the temperature and stress field in SLM. The authors consider powder properties to couple the effect of process parameters on the temperature field distribution with the melt-pool size and the induced thermal stresses.

Leitz et al. [47] used the COMSOL package to simulate the multi-physics FE model for SLM-AM technique. The model includes multi-physics phenomena such as absorption of laser radiation on the surface, conductive and convective heat transfer in the product and the ambient atmosphere, melting, solidification, evaporation, and condensation. Results are validated against experimental data for SLM of steel and molybdenum. A smaller melt pool was observed for molybdenum as it has a higher thermal conductivity than steel. 

**Figure 6:**
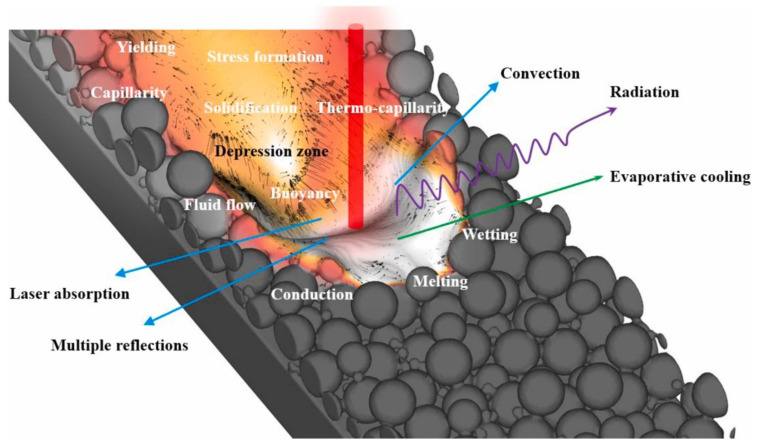
Representative snapshot of the simulation using the Comprehensive Modeling Framework that incorporates within the model different physical phenomena on the metal surface [147].

Li et al. [31] introduced a novel Comprehensive Modeling Framework (CMF) to study the multi-physics problem in laser PBF. This model integrates thermo-fluid and thermo-mechanical models and validated against the additive manufacture of Ti6Al4V. It was found that the thermal-stress concentration was higher near the pores, cavity, and the melt pool. Figure 6 shows the simulated metal surface with different physical phenomena incorporated into the model [147].

The complexity of these models necessitates leveraging extensive computational resources to simulate the different aspects of the thermo-mechanical process. Therefore, results from large-scale simulations of this kind are limited, and rather such models find greater scope in mimicking the AM process in extremely small domains (mm^3^) and time scales (ms) to render results in a reasonable time [57,130,148].

### 2.2. Microstructural Models

**Figure 7:**
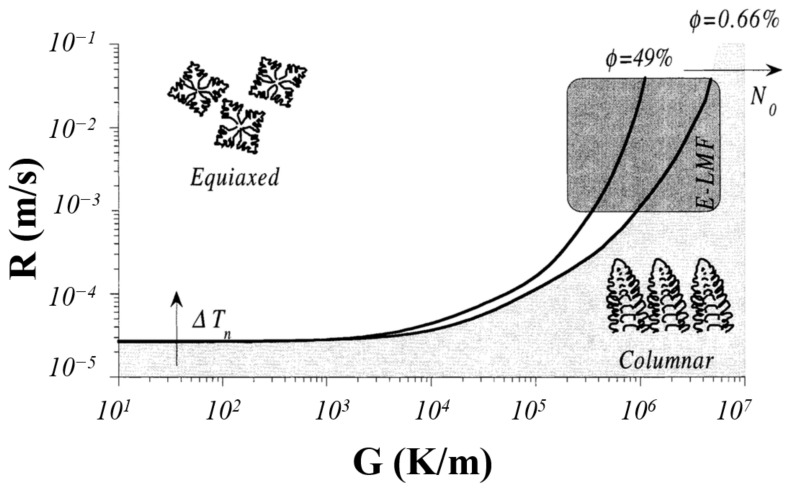
Analysis of the thermal gradient (G) as a function of the solidification rate (R) [150].

Understanding the grain-growth mechanism by analyzing the temperature profiles enables microstructure control to tailor the mechanical properties or produce directionally solidified (DS) and single-crystal (SX) components [93]. Formation of a new surface during solidification requires a significant energy change accompanied by the time for the phase transformation. However, the rapid cooling (often through the substrate and the previously deposited layers) during printing denies both conditions favoring heterogenous nucleation, with the grains originating from the nucleation sites beneath the solid–liquid interface of the melt pool. Post nucleation, grains grow in the direction opposite to the heat transfer driven by the thermal gradient (*G*) and the solidification rate (*R*) [149]. A high *G*/*R* ratio advocates the formation of columnar growth, noted in the melt-pool core, while a reduced *G*/*R* favors equiaxed grains at the top of the melt pool, as displayed in Figure 7 [150]. The cooling rate is defined as *G × R* and is responsible for grain size, suggesting finer grains are observed for higher cooling rates [62]. However, areas of the melt pool exposed to the atmosphere realize equiaxed grains due to convection and radiation from the surroundings. Moreover, scan patterns for PBF and DED are reported to exert a great influence on the grain structures [151]. 

Recent models describing the microstructures in AM that include elementary cellular automata-finite element (CA-FE) [63] and Lattice Boltzmann-cellular automata [64] are primarily used to predict the solidification front with limited effects from subsequent reheating and re-melting. The CA model was developed for the casting process and has been applied to study the microstructural growth (dendrite formation) in AM processes. The stochastic CA approach incorporates nucleation, growth, and diffusion of constituent elements and phases to predict the grain orientations originating in the melt pool. Pauza et al. [152] included the crystallographic orientation information in a Monte-Carlo Potts model for microstructure evolution in a PBF process. Phase field (PF) models, on the other hand, predict the 2D crystal growth dynamics (microstructure evolution) during solidification [62]. The simulation of microstructure evolution in AM has been carried out using the cellular-automata (CA) method and phase-field (PF) methods. The PF method is a very powerful tool to simulate the microstructure evolution in the AM process. Liu et al. [153] studied the effects of process parameters on the morphologies of the melt pool and grain-growth in the PBF-AM process and developed a 2D phase-field model. Further, they modified the model into a 3D PF model and nucleation phenomena was integrated with it to study the columnar to equiaxed transition (CET) in a single-track PBF process. Liquid–solid phase change and grain nucleation, growth, and coarsening in solid regions were included in the PF model to simulate the grain growth during the PBF process. The thermal fluid flow (TFF) [154] model was coupled with the PF model for the temperature profile. Sahoo and Chou [93] developed a PF model to predict the microstructure evolution of Ti-6Al-4V in electron beam AM. They observed in the simulation that the spacing between the columnar dendrites and the width of the dendrites is lower for higher temperature gradient and the electron-beam scanning speed. Nevertheless, limited methods have been developed to replicate the fine-scale microstructural details across a sufficiently large scale to predict the microstructure over many passes and layers [149]. Table 5 lists the parameters and identifies the advantages of several existing models. For predicting the resultant phases post solidification, thermodynamic models like calculation of phase diagrams (CALPHAD) coupled with Scheil-based solidification simulations have proven useful to determine the potential phases that are observed in AM fabricated alloys. Fostering rapid alloy discovery through AM, CALPHAD simulations can assist in forecasting the plausible phase space to design new alloys with limited experiments [155]. The main challenge is the availability of diverse alloy materials for AM. Moreover, novel strategies are adopted to design new alloys to overcome this limitation. By integrating microstructural refinement (MR) and eutectic solidification (ES) in an alloy design strategy, one can circumvent the issue across the various solidification stages. Such integrated MR and ES alloy design strategies enable widening of the alloy-processing window as well as activation of several deformation mechanisms, e.g., back-stress strengthening and work hardening, which produce alloys that exhibit a remarkable synergy between printability and performance. Additionally, by using Olson’s systems approach, one can devise an effective means to integrate the computational material-engineering framework into alloy design for laser-powder bed-fusion AM [156].

### 2.3. Multi-Scale Model

AM is a multi-physical as well as multi-scale process, which involves macro-scale manufacturing and microstructure evolution. In the AM process, stress field and temperature field models are based on the macro scale, flow field models on the melt pool are based on the meso scale, and the material microstructure evolution are based on micro-scale [62,160]. In micro and meso scales, the time scale is in the order of microseconds, but the associated simulations are computer intensive [57]. Moreover, the time and length domains for the processing and microstructure transformation being orders of magnitude displaced, coupling across material and manufacturing scales is a major challenge [57,77,89]. 

Chen et al. [161] used a molecular-dynamics (MD) simulation to study the formation of a medium entropy alloy at the atomic scale in the selective laser melting process. Li et al. [79] studied the effect of scanning speed and laser power on the thermal behavior in SLM and observed that the process parameters exert a significant impact on the temperature distribution, size of melt pool, and microstructure [128,162]. However, the evolution of SLM deposited material has not been examined on the nanoscale that could offer more insight on the structure transformation [163].

Nie et al. [164] developed a multi-scale model by coupling the FEM model with stochastic analysis for IN718 where the temperature field was modeled using FEM and stochastic analysis was employed to evaluate microstructure evolution during solidification. The simulation results were in agreement with the experimentally reported results. 

### 2.4. Machine Learning in AM

AM holds promise for fabrication of multi-functional multi-material components with complex geometries, but its reach is restricted due to challenges such as incompatible properties of materials, non-uniformity, and imperfections in the build part [165,166,167]. To eliminate these challenges, machine-learning (ML) algorithms are being employed to detect the anomaly and optimize the process parameters. ML methods are implemented mainly in three stages of the AM process, viz., design of the product geometry, modulation of the process parameters, and in situ anomaly detection [168]. The main aim of using ML methods in AM is to transform the manufacturing process to be more advanced, efficient, and cost effective. 

The AM process starts with designing the geometry of the product by using computer-aided design (CAD) software. This CAD model is then fed into the 3D printer to fabricate the product. During part printing, the process parameters need to be set appropriately to obtain the desired product features and properties. Generally, these parameters are controlled manually as per the design and condition of the part, which leads to various defects in the final product [168]. Significant research has been carried out to resolve these issues by optimizing the AM process with the help of simulation and ML methods. Simulations and numerical models are used in AM to explore and examine the effects of combining different process parameters [43,169], while ML methods aid in studying the effect of process parameters on the quality of the final product [170,171]. 

ML methods use previous input dataset to generate rules and learning principles to correlate the processing-structure property of the printed part. Mainly three types of ML algorithms are employed, viz., supervised learning, unsupervised learning, and reinforcement learning (RL) [38,168]. In supervised learning, labeled training data are used to construct the model; examples include support vector machines (SVM) and Gaussian processes (GP). These methods are most suited for classification and regression problems [172,173]. The most widely used models are artificial neural networks (ANNs) inspired from the human neural networks to learn and improve their accuracy over time from the training dataset [174]. On the other hand, unsupervised learning is useful in cases where no labeled dataset is available, with clustering and self-organizing maps (SOMs) being two of the popular methods [175]. RL is based on the outcome of an action in the state of the surroundings to achieve the desired outcome. This method is generally used in robotic cars and self-driving vehicles [176].

The AM process workflow starts with designing the part, which needs to be optimized to minimize the number of overhang structures. The latter need support structures during fabrication to restrain the part in place, and after fabrication those support structures are removed manually, a tedious and time-consuming process [168]. Topology optimization (TO) is implemented in AM to efficiently design the desired part with given constraint across length scales [177,178]. AM has capabilities to build a complex lattice structure, which can be represented as a digital (twin) material (DM). This concept is used to represent the complex lattice structure, where the DM is a group of voxels (a lattice of a material element) [179]. The DM is integrated with ML to predict the toughness, strength, and deformation of the material where the voxels of the DM is used as an input dataset to ML to enable an efficient and cost-effective design. 

The most important step to print a part by AM is to set the process parameters precisely to obtain the desired defect-limited product. A large number of process parameters are associated with AM; the combination of these parameters needs optimization. A combinatorial process-parameter optimization by experiments is time and resource intensive; computational models can be material and product scale-specific and depending on the complexity may be computationally demanding or infeasible. Data-driven ML methods can help in alleviating these limitations to enable efficient, faster, and accurate predictions. Convolution Neural Network (CNN) has been used to predict the print quality in FFF (fused filament fabrication) for various parameters such as print speed, fan speed, and extrusion multiplier [170]. NNs were also used to determine the geometrical inaccuracies in the part generated by the residual stress. Kappes et al. [180] introduced a ML model by which porosity in the part could be determined based on the print orientation. The consequence of part position, print orientation on the keyhole formation, and lack of fusion can be determined using ML models. However, to detect in situ defects, a continuous and synchronous monitoring system is required that leverage image processing and ML. In FFF, a DIC (digital image correlation) camera is installed to monitor the surface geometry. From the stereoscopic image, the surface geometry is reconstructed using a random sample consensus (RANSAC) algorithm [181]. This method is used for alignment of the parts and is useful for porosity detection. The training data for CNN models are also used to detect the in-situ anomaly from the images and reconstruct the geometry by varying the process parameters. The accuracy of the printed part is noted to increase significantly by integration of ML. 

Computer vision is emerging as the next-generation tool to predict and control quality of AM parts, and to enhance production planning in cloud-based AM platforms. Through deep learning and integration of visual features, this approach aims to optimize production processes, ensuring efficient resource allocation and improved manufacturing outcomes [182]. During the powder deposition stage of the process, an automated computer vision algorithm is employed for the detection and categorization of anomalies. This toolkit involves the application of an unsupervised machine-learning algorithm on a moderately sized training dataset of image patches to enable effective anomaly detection and in situ optimization [183]. Moreover, computer-vision approaches can be used for quality control of parts fabricated using AM [184], as well as to predict the powder flowability in metal AM [185]. Different ML algorithms, listed in Table 6, can be implemented at various stages of AM guided by the adaptability of the predictive techniques.

## 3. Outlook

With process parameters being predominantly responsible for the undesired artifacts such as porosities, key holes, balling, residual stresses, and cracks, there is a need to understand the effects of these process variables on the resultant quality of the AM fabricated parts. However, experimental investigations to interrogate these effects are resource and time intensive, necessitating the ever-increasing role of digital experiments via accurate numerical models. Nevertheless, the computational cost and the associated degree of complexity that can be examined through predictive models for a mimicry of the physical processing, continues to be a challenge. Complications in the models range from the adoption of complex discretization techniques to the inclusion of fluid-flow effects like the Marangoni phenomenon within the melt pool. For instance, a typical practice for discretization of geometry considers finer elements to describe areas under high stress (points of interest), and modeling the areas away from the points of interest with coarser grids. Notably, model complexities also increase the computational costs. Apart from transport mechanisms, i.e., heat transfer and fluid flow, material properties, viz., microstructures, residual stresses, dislocation, and grain boundaries, can also be represented via various levels of fidelities, necessitating an intelligent selection based on a tradeoff between computer time and accuracy. 

Over the last decade, machine-learning (ML) models and computer vision have emerged as viable tools to assist in the choice of the appropriate computational models and more recently, offering predictability for AM processes. Although ML for AM can guide the initial processing parameter window for optimal and certifiable AM parts, these models rely on the data available for their training, thus establishing a stronger case for AM data curation, storage, and dissemination for re-use. Given that AM is an inherently stochastic process with appreciable associated uncertainties in material properties, manufacturing conditions and environmental variables, repeatability, and reproducibility of part production requires performing extensive experiments for statistical averaging. To account for such challenges, uncertainty quantification (UQ) can offer valuable insights on the effects of processing parameters on the part geometry, temperature profiles, properties realized, and provide information on the selection of processing parameters to achieve targeted component specifications. 

Scalable AM-based fabrication poses an even greater challenge. Scaled-up simulations at the increased length and time scales are uber computer intensive and may even require longer computational wall times than an actual experiment. However, given the material, equipment, and personnel costs associated with scaled-up powder AM platforms, efficient predictive models with minimal compromise in accuracy are imminent to determine features such as residual stresses and material properties, and to implement an optimal and economic AM process control. 

For AM components to deliver mission-critical performance, the parts must exhibit the desired microstructure; this in turn is highly dependent on the nature, severity, and concentration of processing related defects. Consequently, AM process qualification and part certification, for defect-formation control, including that due to instrument sensitivity, is imperative. Specially, the following challenges persist: (a) given that AM for engineering applications needs to produce fail-safe components, full-load condition testing, even for conservative builds, is expensive; and (b) certification based on allowable processing metrics to manufacture parts within threshold defect criteria require testing and microstructural characterization of coupons that may take months to complete. Hence, a model centric paradigm is inevitable to overcome these drawbacks and realize the promise that AM holds.

In the realm of rapid predictive certification for AM, the goal of future efforts should be to construct a high-throughput and data-informed qualification paradigm for defect detection at the instance of occurrence such that suitable mitigation or cessation of the AM process can be implemented on demand. To realize this goal, the research objectives can be two-fold: fundamentally, understand *what the root causes for a defect during AM of metals and alloys are,* and *identify processing parameter windows, portable across machines, to prevent and minimize anomalies*. The objectives can be achieved through a synergy of (1) robust data analytics of exhaustive and varied in situ process characterizations, (2) high-throughput evaluations to validate materials-process-structure-property predictions derived from multiscale computational toolkits, and (3) rigorous UQ of models and Bayesian calibration of parameters from in situ and property measurements for fabricating defect-limited and performance-safe parts. Critically, future efforts should capitalize on industrial partnerships for scalable part manufacture and testing to validate model-centric standardization frameworks. An in-situ data guided paradigm will not only expedite and cheapen AM part fabrication but will additionally streamline environmental testing under operational conditions.

## Figures and Tables

**Table 1:** Build rates and surface roughness of AM processes illustrated in Figure 1 [1,12].

AM Technique	Build Rate (cm^3^/h)	Surface Roughness (µm)
Selective Laser Melting (SLM) [19]	~70	4–11
Laser Metal Deposition (LMD) [20]	~300	10–200

**Table 2:** Comparison of runtime between adaptive mesh and fine mesh [51].

Mesh Scheme	Layer Number/ Runtime (h)						Total Runtime (h)
Adaptive mesh (coarsening approach)	1–3	4–6	7–9	10–12	13–15	16–18	20:10 + 2:00 (extra 2 h for mapping)
	1:50	2:35	3:20	3:50	4:10	4:20	
Fine mesh	1–18						58:30 (no mapping required)
	58:30						

**Table 3:** Key parameters required to simulate PBF process [7].

Process Parameters	Type/Unit
Heat source type	Goldak’s Ellipsoidal/Gaussian distribution
Power input	Watt
Deposition layer thickness	Micron (µm)
Hatch spacing	µm
Each layer printing time	Second (s)
Idle time between two layers	s
Scanning pattern type	Uni/bi/cross-directional, island, helix
Scanning laser speed	mm/s
Ambient/Pre-heat temperature	Celsius/Kelvin

**Table 4:** Effects of various impact factors on the fabricated AM component [7].

Impact Factors	Effects
Geometry of the base plate [132]	The residual stress is uniform and lower for thick base plate.
Base plate and build chamber pre-heating [132,143]	Residual stress can be decreased by using preheated build chamber, which reduces temperature gradient.
Orientation of the build part [144,145]	Residual stress is minimum for particular build orientation
Support structure for build part [49]	Distortion can be reduced by using proper support.
Scanning sequence [102]	Residual stress reduced by applying proper fabrication sequence.
Scanning pattern [28,102,146]	In case of fractal, spiral, and small-piece scanning patterns, the stress reduced.
Scanning power and speed [102,146]	Rate of change in strain is higher for higher energy density.
Scanning length (direction) [102,146]	Higher residual stress is generated for long scanning vector as it causes large temperature gradient.
Addition of layers [132]	Residual stress increases for higher number of layers.
Layer of the geometry [146]	Residual stress varies with different geometry shape and their accumulation.
Deposition layer thickness [117]	High stress and deformation are generated in case of thin layer.
Temperature gradient [146]	High residual stress is generated due to high temperature gradient and higher cooling rate.

**Table 5:** Advantages and disadvantages of different AM microstructure simulation methods [149].

Methods	Advantages	Disadvantages
Empirical microstructure modeling [157]	Microstructural attributes for large builds can be predicted. It allows extension of pre-existing thermal models. The computational cost is low if thermal results exist, otherwise medium.	Microstructure for further investigation is not provided. The thermal environment estimation is required for analysis.
Monte Carlo [158]	With hundreds of heat source passes, it can predict entire 3D microstructures. In the course of solidification and solid-state grain evolution, it can provide an approximation of micro-structure. Without the requirement to parameterize for distinct material systems, it uses idealized molten zones. Open-source SPPARKS Monte Carlo suite includes it.	Direct coupling of thermal and microstructural models cannot be completed. It does not currently take material texture or anisotropy into account. Computational cost is medium.
Cellular Automata—Lattice Boltzmann [64]	It can be applied to the coupled evolution of microstructure and thermo-fluid on the same lattice. The crystallographic texture is incorporated here.	Sometimes unstable solutions can be seen for many regimes. Solid-state grain evolution cannot be simulated after solidification. Only a few passes of a heat source can be used. The computational cost is high.
Cellular Automata—Finite Element (CAFE) [63,159]	Coupled prediction of thermal behavior and microstructure can be achieved. Moreover, the crystallographic texture is incorporated here also.	Solid-state grain evolution cannot be simulated after solidification. Also, here, only a few passes of a heat source can be used. It has high computational cost.

**Table 6:** List of ML algorithms used in AM processes [168].

AM Processes	ML Algorithms
Optimization of geometry	Clustering, Neural Networks (NN) and Support Vector Machines (SVM) [186,187]
Design of material	Convolutional Neural Network (CNN) and Decision trees [186,187]
Determination of process parameter	Neural Networks (NN) and Principal component analysis (PCA) [188]
Defects identification	Clustering, Convolutional Neural Network (CNN), and Support Vector Machines (SVM) [171,172]
Quality assessment	Convolutional Neural Network (CNN), Self-organizing map (SOM), and Gaussian processes (GP) [173,189]

## Data Availability

The data presented in this study are available on request from the corresponding author.

## References

[1] Gorsse S., Hutchinson C., Gouné M., Banerjee R. (2017). Additive Manufacturing of Metals: A Brief Review of the Characteristic Microstructures and Properties of Steels, Ti-6Al-4V and High-Entropy Alloys. Sci. Technol. Adv. Mater..

[2] Martin B.W., Ales T.K., Rolchigo M.R., Collins P.C. (2019). Developing and Applying ICME + Modeling Tools to Predict Performance of Additively Manufactured Aerospace Parts. Additive Manufacturing for the Aerospace Industry.

[3] Slotwinski J.A. (2014). Additive Manufacturing: Overview and NDE Challenges. AIP Conf. Proc..

[4] (2013). ISO/ASTM Additive Manufacturing—General Principles Terminology (ASTM52900).

[5] Tapia G., Elwany A. (2014). A Review on Process Monitoring and Control in Metal-Based Additive Manufacturing. J. Manuf. Sci. Eng..

[6] Ha K., Kim T., Baek G.Y., Jeon J.B., Shim D., Moon Y.H., Lee W. (2020). Numerical Study of the Effect of Progressive Solidification on Residual Stress in Single-Bead-on-Plate Additive Manufacturing. Addit. Manuf..

[7] Luo Z., Zhao Y. (2018). A Survey of Finite Element Analysis of Temperature and Thermal Stress Fields in Powder Bed Fusion Additive Manufacturing. Addit. Manuf..

[8] Lores A., Azurmendi N., Agote I., Zuza E. (2019). A Review on Recent Developments in Binder Jetting Metal Additive Manufacturing: Materials and Process Characteristics. Powder Metall..

[9] Li M., Du W., Elwany A., Pei Z., Ma C. (2020). Metal Binder Jetting Additive Manufacturing: A Literature Review. J. Manuf. Sci. Eng..

[10] Charts B., Bhagavatam A., Sreeramagiri P., Dinda G., Solutions A.P., Creek C. (2020). Microstructure and Mechanical Properties of Direct Laser Metal Deposited GRCop-84 Alloy.

[11] Attaran M. (2017). The Rise of 3-D Printing: The Advantages of Additive Manufacturing over Traditional Manufacturing. Bus. Horiz..

[12] Wong K.V., Hernandez A. (2012). A Review of Additive Manufacturing. ISRN Mech. Eng..

[13] Kamal M., Rizza G. (2019). Design for Metal Additive Manufacturing for Aerospace Applications. Additive Manufacturing for the Aerospace Industry.

[14] Murr L.E. (2018). Additive Manufacturing of Biomedical Devices: An Overview. Mater. Technol..

[15] Wang C., Tan X., Liu E., Tor S.B. (2018). Process Parameter Optimization and Mechanical Properties for Additively Manufactured Stainless Steel 316L Parts by Selective Electron Beam Melting. Mater. Des..

[16] Cheng B., Price S., Lydon J., Cooper K., Chou K. (2014). On Process Temperature in Powder-Bed Electron Beam Additive Manufacturing: Model Development and Validation. J. Manuf. Sci. Eng..

[17] Li C., Fu C.H., Guo Y.B., Fang F.Z. (2015). Fast Prediction and Validation of Part Distortion in Selective Laser Melting. Procedia Manuf..

[18] Sreeramagiri P., Balasubramanian G. (2022). A Process Parameter Predictive Framework for Laser Cladding of Multi-Principal Element Alloys. Addit. Manuf. Lett..

[19] Gibson I., Rosen D.W., Stucker B. (2010). Additive Manufacturing Technologies.

[20] Seifi M., Salem A., Beuth J., Harrysson O., Lewandowski J.J. (2016). Overview of Materials Qualification Needs for Metal Additive Manufacturing. JOM.

[21] Zhang H., Pan Y., He Y.Z., Wu J.L., Yue T.M., Guo S. (2014). Application Prospects and Microstructural Features in Laser-Induced Rapidly Solidified High-Entropy Alloys. JOM.

[22] Matsumoto M., Shiomi M., Osakada K., Abe F. (2002). Finite Element Analysis of Single Layer Forming on Metallic Powder Bed in Rapid Prototyping by Selective Laser Processing. Int. J. Mach. Tools Manuf..

[23] Lindgren L.-E., Hedblom E. (2001). Modelling of Addition of Filler Material in Large Deformation Analysis of Multipass Welding. Commun. Numer. Methods Eng..

[24] Svetlizky D., Das M., Zheng B., Vyatskikh A.L., Bose S., Bandyopadhyay A., Schoenung J.M., Lavernia E.J., Eliaz N. (2021). Directed Energy Deposition (DED) Additive Manufacturing: Physical Characteristics, Defects, Challenges and Applications. Mater. Today.

[25] Wu A.S., Brown D.W., Kumar M., Gallegos G.F., King W.E. (2014). An Experimental Investigation into Additive Manufacturing-Induced Residual Stresses in 316L Stainless Steel. Metall. Mater. Trans. A.

[26] Childs T.H.C., Hauser C., Badrossamay M. (2005). Selective Laser Sintering (Melting) of Stainless and Tool Steel Powders: Experiments and Modelling. Proc. Inst. Mech. Eng. Part B J. Eng. Manuf..

[27] Denlinger E.R., Heigel J.C., Michaleris P. (2015). Residual Stress and Distortion Modeling of Electron Beam Direct Manufacturing Ti-6Al-4V. Proc. Inst. Mech. Eng. Part B J. Eng. Manuf..

[28] Rivalta F., Ceschini L., Jarfors A.E.W., Stolt R. (2021). Effect of Scanning Strategy in the L-PBF Process of 18Ni300 Maraging Steel. Metals.

[29] Miranda G., Faria S., Bartolomeu F., Pinto E., Madeira S., Mateus A., Carreira P., Alves N., Silva F.S., Carvalho O. (2016). Predictive Models for Physical and Mechanical Properties of 316L Stainless Steel Produced by Selective Laser Melting. Mater. Sci. Eng. A.

[30] Laakso P., Riipinen T., Laukkanen A., Andersson T., Jokinen A., Revuelta A., Ruusuvuori K. (2016). Optimization and Simulation of SLM Process for High Density H13 Tool Steel Parts. Phys. Procedia.

[31] Li W., Nagaraja K.M., Zhang X., Zhou R., Qian D., Lu H. (2022). Multi-Physics Modeling of Powder Bed Fusion Process and Thermal Stress near Porosity. Manuf. Lett..

[32] Antony K., Arivazhagan N., Senthilkumaran K. (2014). Numerical and Experimental Investigations on Laser Melting of Stainless Steel 316L Metal Powders. J. Manuf. Process..

[33] Küng V.E., Scherr R., Markl M., Körner C. (2021). Multi-Material Model for the Simulation of Powder Bed Fusion Additive Manufacturing. Comput. Mater. Sci..

[34] Fu C.H., Guo Y.B. (2014). Three-Dimensional Temperature Gradient Mechanism in Selective Laser Melting of Ti-6Al-4V. J. Manuf. Sci. Eng..

[35] Dong L., Correia J.P.M., Barth N., Ahzi S. (2017). Finite Element Simulations of Temperature Distribution and of Densification of a Titanium Powder during Metal Laser Sintering. Addit. Manuf..

[36] Lundbäck A., Lindgren L.E. (2011). Modelling of Metal Deposition. Finite Elem. Anal. Des..

[37] Marrey M., Malekipour E., El-Mounayri H., Faierson E.J. (2019). A Framework for Optimizing Process Parameters in Powder Bed Fusion (PBF) Process Using Artificial Neural Network (ANN). Procedia Manuf..

[38] Meng L., McWilliams B., Jarosinski W., Park H.-Y., Jung Y.-G., Lee J., Zhang J. (2020). Machine Learning in Additive Manufacturing: A Review. JOM.

[39] Lindgren L.-E., Runnemalm H., Nasstrom M.O. (1999). Simulation of Multipass Welding of a Thick Plate. Int. J. Numer. Methods Eng..

[40] Ales T.K. (2018). An Integrated Model for the Probabilistic Prediction of Yield Strength in Electron-Beam Additively Manufactured Ti-6Al-4V.

[41] Ghamarian I., Hayes B., Samimi P., Welk B.A., Fraser H.L., Collins P.C. (2016). Developing a Phenomenological Equation to Predict Yield Strength from Composition and Microstructure in β Processed Ti-6Al-4V. Mater. Sci. Eng. A.

[42] King W.E., Anderson A.T., Ferencz R.M., Hodge N.E., Kamath C., Khairallah S.A., Rubenchik A.M. (2015). Laser Powder Bed Fusion Additive Manufacturing of Metals; Physics, Computational, and Materials Challenges. Appl. Phys. Rev..

[43] Raghavan N., Dehoff R., Pannala S., Simunovic S., Kirka M., Turner J., Carlson N., Babu S.S. (2016). Numerical Modeling of Heat-Transfer and the Influence of Process Parameters on Tailoring the Grain Morphology of IN718 in Electron Beam Additive Manufacturing. Acta Mater..

[44] Gu D., He B. (2016). Finite Element Simulation and Experimental Investigation of Residual Stresses in Selective Laser Melted Ti-Ni Shape Memory Alloy. Comput. Mater. Sci..

[45] Bontha S., Klingbeil N.W., Kobryn P.A., Fraser H.L. (2006). Thermal Process Maps for Predicting Solidification Microstructure in Laser Fabrication of Thin-Wall Structures. J. Mater. Process. Technol..

[46] Huang X., Chen H., Liu B., Mohammadzadeh R., Li J., Fang Q. (2021). Thermal Behavior and Microstructural Evolution of Additively Manufactured Ni-Based Superalloys via Multi-Scale Simulation. Optik.

[47] Leitz K.H., Singer P., Plankensteiner A., Tabernig B., Kestler H., Sigl L.S. (2017). Multi-Physical Simulation of Selective Laser Melting. Met. Powder Rep..

[48] Van Belle L., Vansteenkiste G., Boyer J.C. (2012). Comparisons of Numerical Modelling of the Selective Laser Melting. Key Eng. Mater..

[49] Hussein A., Hao L., Yan C., Everson R. (2013). Finite Element Simulation of the Temperature and Stress Fields in Single Layers Built Without-Support in Selective Laser Melting. Mater. Des..

[50] Childs T.H.C., Berzins M., Ryder G.R., Tontowi A. (1999). Selective Laser Sintering of an Amorphous Polymer—Simulations and Experiments. Proc. Inst. Mech. Eng. Part B J. Eng. Manuf..

[51] Hajializadeh F., Ince A. (2019). Finite Element–Based Numerical Modeling Framework for Additive Manufacturing Process. Mater. Des. Process. Commun..

[52] Zeng K., Pal D., Gong H.J., Patil N., Stucker B. (2015). Comparison of 3DSIM Thermal Modelling of Selective Laser Melting Using New Dynamic Meshing Method to ANSYS. Mater. Sci. Technol..

[53] Pal D., Patil N., Nikoukar M., Zeng K., Kutty K.H., Stucker B.E. An Integrated Approach to Cyber-Enabled Additive Manufacturing Using Physics Based, Coupled Multi-Scale Process Modeling. Proceedings of the 24th International SFF Symposium—An Additive Manufacturing Conference 2013.

[54] Gouge M., Michaleris P., Denlinger E., Irwin J. (2018). The Finite Element Method for the Thermo-Mechanical Modeling of Additive Manufacturing Processes. Thermo-Mechanical Modeling of Additive Manufacturing.

[55] Zhang D.Q., Cai Q.Z., Liu J.H., Zhang L., Li R.D. (2010). Select Laser Melting of W–Ni–Fe Powders: Simulation and Experimental Study. Int. J. Adv. Manuf. Technol..

[56] Roberts I.A., Wang C.J., Esterlein R., Stanford M., Mynors D.J. (2009). A Three-Dimensional Finite Element Analysis of the Temperature Field during Laser Melting of Metal Powders in Additive Layer Manufacturing. Int. J. Mach. Tools Manuf..

[57] Bugatti M., Semeraro Q. (2018). Limitations of the Inherent Strain Method in Simulating Powder Bed Fusion Processes. Addit. Manuf..

[58] Patil R.B., Yadava V. (2007). Finite Element Analysis of Temperature Distribution in Single Metallic Powder Layer during Metal Laser Sintering. Int. J. Mach. Tools Manuf..

[59] Dai D., Gu D. (2014). Thermal Behavior and Densification Mechanism during Selective Laser Melting of Copper Matrix Composites: Simulation and Experiments. Mater. Des..

[60] Wang J., Wang Y., Shi J. (2021). A Novel Time Step Fusion Method with Finite Volume Formulation for Accelerated Thermal Analysis of Laser Additive Manufacturing. Int. J. Precis. Eng. Manuf. Technol..

[61] Stump B., Plotkowski A., Coleman J. (2021). Solidification Dynamics in Metal Additive Manufacturing: Analysis of Model Assumptions. Model. Simul. Mater. Sci. Eng..

[62] Liu C., Gao H., Li L., Wang J., Guo C., Jiang F. (2021). A Review on Metal Additive Manufacturing: Modeling and Application of Numerical Simulation for Heat and Mass Transfer and Microstructure Evolution. China Foundry.

[63] Zinoviev A., Zinovieva O., Ploshikhin V., Romanova V., Balokhonov R. (2016). Evolution of Grain Structure during Laser Additive Manufacturing. Simulation by a Cellular Automata Method. Mater. Des..

[64] Rai A., Markl M., Körner C. (2016). A Coupled Cellular Automaton–Lattice Boltzmann Model for Grain Structure Simulation during Additive Manufacturing. Comput. Mater. Sci..

[65] Rolchigo M.R., LeSar R. (2018). Modeling of Binary Alloy Solidification under Conditions Representative of Additive Manufacturing. Comput. Mater. Sci..

[66] Lu L.X., Sridhar N., Zhang Y.W. (2018). Phase Field Simulation of Powder Bed-Based Additive Manufacturing. Acta Mater..

[67] Fleck M., Querfurth F., Glatzel U. (2017). Phase Field Modeling of Solidification in Multi-Component Alloys with a Case Study on the Inconel 718 Alloy. J. Mater. Res..

[68] Paul R., Anand S., Gerner F. (2014). Effect of Thermal Deformation on Part Errors in Metal Powder Based Additive Manufacturing Processes. J. Manuf. Sci. Eng..

[69] Kumar S. (2003). Selective Laser Sintering: A Qualitative and Objective Approach. JOM.

[70] Elsayed M., Ghazy M., Youssef Y., Essa K. (2019). Optimization of SLM Process Parameters for Ti6Al4V Medical Implants. Rapid Prototyp. J..

[71] Ahmadi A., Mirzaeifar R., Moghaddam N.S., Turabi A.S., Karaca H.E., Elahinia M. (2016). Effect of Manufacturing Parameters on Mechanical Properties of 316L Stainless Steel Parts Fabricated by Selective Laser Melting: A Computational Framework. Mater. Des..

[72] Calignano F. (2018). Investigation of the Accuracy and Roughness in the Laser Powder Bed Fusion Process. Virtual Phys. Prototyp..

[73] Yadroitsev I., Gusarov A., Yadroitsava I., Smurov I. (2010). Single Track Formation in Selective Laser Melting of Metal Powders. J. Mater. Process. Technol..

[74] Raghavan N., Simunovic S., Dehoff R., Plotkowski A., Turner J., Kirka M., Babu S. (2017). Localized Melt-Scan Strategy for Site Specific Control of Grain Size and Primary Dendrite Arm Spacing in Electron Beam Additive Manufacturing. Acta Mater..

[75] Liverani E., Toschi S., Ceschini L., Fortunato A. (2017). Effect of Selective Laser Melting (SLM) Process Parameters on Microstructure and Mechanical Properties of 316L Austenitic Stainless Steel. J. Mater. Process. Technol..

[76] Moradi M., Hasani A., Pourmand Z., Lawrence J. (2021). Direct Laser Metal Deposition Additive Manufacturing of Inconel 718 Superalloy: Statistical Modelling and Optimization by Design of Experiments. Opt. Laser Technol..

[77] Markl M., Körner C. (2016). Multiscale Modeling of Powder Bed–Based Additive Manufacturing. Annu. Rev. Mater. Res..

[78] Gibson I., Rosen D., Stucker B. (2010). Additive Manufacturing Technologies—Rapid Prototyping.

[79] Li Y., Gu D. (2014). Parametric Analysis of Thermal Behavior during Selective Laser Melting Additive Manufacturing of Aluminum Alloy Powder. Mater. Des..

[80] Tran T.Q., Chinnappan A., Lee J.K.Y., Loc N.H., Tran L.T., Wang G., Kumar V.V., Jayathilaka W.A.D.M., Ji D., Doddamani M. (2019). 3D Printing of Highly Pure Copper. Metals.

[81] Gatsos T., Elsayed K.A., Zhai Y., Lados D.A. (2020). Review on Computational Modeling of Process–Microstructure–Property Relationships in Metal Additive Manufacturing. JOM.

[82] Raghavan A., Wei H.L., Palmer T.A., DebRoy T. (2013). Heat Transfer and Fluid Flow in Additive Manufacturing. J. Laser Appl..

[83] Chatterjee A., Kumar S., Saha P., Mishra P., Choudhury A.R. (2003). An Experimental Design Approach to Selective Laser Sintering of Low Carbon Steel. J. Mater. Process. Technol..

[84] DebRoy T., Wei H.L., Zuback J.S., Mukherjee T., Elmer J.W., Milewski J.O., Beese A.M., Wilson-Heid A., De A., Zhang W. (2018). Additive Manufacturing of Metallic Components—Process, Structure and Properties. Prog. Mater. Sci..

[85] King W.E., Barth H.D., Castillo V.M., Gallegos G.F., Gibbs J.W., Hahn D.E., Kamath C., Rubenchik A.M. (2014). Observation of Keyhole-Mode Laser Melting in Laser Powder-Bed Fusion Additive Manufacturing. J. Mater. Process. Technol..

[86] Sames W.J., Medina F., Peter W.H., Babu S.S., Dehoff R.R. (2014). Effect of Process Control and Powder Quality on Inconel 718 Produced Using Electron Beam Melting, Proceedings of the 8th International Symposium on Superalloy 718 and Derivatives, Pittsburgh, PA, USA, 29 September–1 October 2014.

[87] Darvish K., Chen Z.W., Pasang T. (2016). Reducing Lack of Fusion during Selective Laser Melting of CoCrMo Alloy: Effect of Laser Power on Geometrical Features of Tracks. Mater. Des..

[88] Mukherjee T., Zuback J.S., De A., DebRoy T. (2016). Printability of Alloys for Additive Manufacturing. Sci. Rep..

[89] Wei H.L., Mukherjee T., Zhang W., Zuback J.S., Knapp G.L., De A., DebRoy T. (2021). Mechanistic Models for Additive Manufacturing of Metallic Components. Prog. Mater. Sci..

[90] Schoinochoritis B., Chantzis D., Salonitis K. (2017). Simulation of Metallic Powder Bed Additive Manufacturing Processes with the Finite Element Method: A Critical Review. Proc. Inst. Mech. Eng. Part B J. Eng. Manuf..

[91] Majumdar T., Bazin T., Massahud Carvalho Ribeiro E., Frith J.E., Birbilis N. (2019). Understanding the Effects of PBF Process Parameter Interplay on Ti-6Al-4V Surface Properties. PLoS ONE.

[92] Matthews M.J., Guss G., Khairallah S.A., Rubenchik A.M., Depond P.J., King W.E. (2016). Denudation of Metal Powder Layers in Laser Powder Bed Fusion Processes. Acta Mater..

[93] Sahoo S., Chou K. (2016). Phase-Field Simulation of Microstructure Evolution of Ti–6Al–4V in Electron Beam Additive Manufacturing Process. Addit. Manuf..

[94] Tsopanos S., Mines R.A.W., McKown S., Shen Y., Cantwell W.J., Brooks W., Sutcliffe C.J. (2010). The Influence of Processing Parameters on the Mechanical Properties of Selectively Laser Melted Stainless Steel Microlattice Structures. J. Manuf. Sci. Eng..

[95] Wang D., Yang Y., Yi Z., Su X. (2013). Research on the Fabricating Quality Optimization of the Overhanging Surface in SLM Process. Int. J. Adv. Manuf. Technol..

[96] Zhang J., Li D.Y., Qiu B., Zhao L.Z. (2011). Simulation of Temperature Field in Selective Laser Sintering on PA6/Cu Composite Powders. Adv. Mater. Res..

[97] LeMay I. (1982). Analysis of Welded Structures. Metallography.

[98] Peyre P., Aubry P., Fabbro R., Neveu R., Longuet A. (2008). Analytical and Numerical Modelling of the Direct Metal Deposition Laser Process. J. Phys. D Appl. Phys..

[99] Cheng B., Shrestha S., Chou K. (2016). Stress and Deformation Evaluations of Scanning Strategy Effect in Selective Laser Melting. Addit. Manuf..

[100] Contuzzi N., Campanelli S.L., Ludovico A.D. (2011). 3D Finite Element Analysis in the Selective Laser Melting Process. Int. J. Simul. Model..

[101] Chen T., Zhang Y. (2007). Three-Dimensional Modeling of Laser Sintering of a Two-Component Metal Powder Layer on Top of Sintered Layers. J. Manuf. Sci. Eng. Trans. ASME.

[102] Dai K., Shaw L. (2001). Thermal and Stress Modeling of Multi-Material Laser Processing. Acta Mater..

[103] Liu F.R., Zhang Q., Zhou W.P., Zhao J.J., Chen J.M. (2012). Micro Scale 3D FEM Simulation on Thermal Evolution within the Porous Structure in Selective Laser Sintering. J. Mater. Process. Technol..

[104] Gaur U., Wunderlich B.B., Wunderlich B. (1983). Heat Capacity and Other Thermodynamic Properties of Linear Macromolecules. VII. Other Carbon Backbone Polymers. J. Phys. Chem. Ref. Data.

[105] Vrancken B., Thijs L., Kruth J.P., Van Humbeeck J. (2012). Heat Treatment of Ti6Al4V Produced by Selective Laser Melting: Microstructure and Mechanical Properties. J. Alloys Compd..

[106] Yamanaka K., Saito W., Mori M., Matsumoto H., Sato S., Chiba A. (2019). Abnormal Grain Growth in Commercially Pure Titanium during Additive Manufacturing with Electron Beam Melting. Materialia.

[107] Shiomi M., Yoshidome A., Abe F., Osakada K. (1999). Finite Element Analysis of Melting and Solidifying Processes in Laser Rapid Prototyping of Metallic Powders. Int. J. Mach. Tools Manuf..

[108] Bugeda G., Cervera M., Lombera G. (1999). Numerical Prediction of Temperature and Density Distributions in Selective Laser Sintering Processes. Rapid Prototyp. J..

[109] Tolochko N.K., Khlopkov Y.V., Mozzharov S.E., Ignatiev M.B., Laoui T., Titov V.I. (2000). Absorptance of Powder Materials Suitable for Laser Sintering. Rapid Prototyp. J..

[110] Hurly J.J. (1999). Thermophysical Properties of Gaseous CF4 and C2F6 from Speed-of-Sound Measurements. Int. J. Thermophys..

[111] Sun M.M., Beaman J.J., Ming S., Sun M.M., Beaman J.J. (2013). A Three Dimensional Model for Selective Laser Sintering.

[112] Rahman M.S., Schilling P.J., Herrington P.D., Chakravarty U.K. (2018). A Comparative Study Between Selective Laser Melting and Electron Beam Additive Manufacturing Based on Thermal Modeling. Volume 1: Advances in Aerospace Technology.

[113] Voller V.R., Brent A.D., Prakash C. (1989). The Modelling of Heat, Mass and Solute Transport in Solidification Systems. Int. J. Heat Mass Transf..

[114] Bai X., Colegrove P., Ding J., Zhou X., Diao C., Bridgeman P., Roman Hönnige J., Zhang H., Williams S. (2018). Numerical Analysis of Heat Transfer and Fluid Flow in Multilayer Deposition of PAW-Based Wire and Arc Additive Manufacturing. Int. J. Heat Mass Transf..

[115] Megahed M., Mindt H.W., N’Dri N., Duan H., Desmaison O. (2016). Metal Additive-Manufacturing Process and Residual Stress Modeling. Integr. Mater. Manuf. Innov..

[116] Foteinopoulos P., Papacharalampopoulos A., Stavropoulos P. (2018). On Thermal Modeling of Additive Manufacturing Processes. CIRP J. Manuf. Sci. Technol..

[117] Singh S.N., Chowdhury S., Nirsanametla Y., Deepati A.K., Prakash C., Singh S., Wu L.Y., Zheng H.Y., Pruncu C. (2021). A Comparative Analysis of Laser Additive Manufacturing of High Layer Thickness Pure Ti and Inconel 718 Alloy Materials Using Finite Element Method. Materials.

[118] Yin J., Zhu H., Ke L., Lei W., Dai C., Zuo D. (2012). Simulation of Temperature Distribution in Single Metallic Powder Layer for Laser Micro-Sintering. Comput. Mater. Sci..

[119] Tian Y.H., Wang C.Q., Zhu D.Y. (2007). Modeling of the Temperature Field during Electron Beam Welding of Aluminum Alloy by a Pre-Defined Keyhole. Key Eng. Mater..

[120] Goldak J., Chakravarti A., Bibby M. (1984). A New Finite Element Model for Welding Heat Sources. Metall. Trans. B.

[121] Irwin J., Michaleris P. (2016). A Line Heat Input Model for Additive Manufacturing. J. Manuf. Sci. Eng..

[122] Fu C.H., Guo Y.B. (2014). 3-Dimensional Finite Element Modeling of Selective Laser Melting TI-6AL-4V Alloy.

[123] Bontha S., Klingbeil N.W., Kobryn P.A., Fraser H.L. (2009). Effects of Process Variables and Size-Scale on Solidification Microstructure in Beam-Based Fabrication of Bulky 3D Structures. Mater. Sci. Eng. A.

[124] Zhao Y., Koizumi Y., Aoyagi K., Wei D., Yamanaka K., Chiba A. (2019). Molten Pool Behavior and Effect of Fluid Flow on Solidification Conditions in Selective Electron Beam Melting (SEBM) of a Biomedical Co-Cr-Mo Alloy. Addit. Manuf..

[125] Semiatin S.L., Ivanchenko V.G., Ivasishin O.M. (2004). Diffusion Models for Evaporation Losses during Electron-Beam Melting of Alpha/Beta-Titanium Alloys. Metall. Mater. Trans. B.

[126] Swain A., Bhattacharya A. Effect of Marangoni and Natural Convection during Laser Melting. Proceedings of the 64th Congress of Indian Society of Theoretical and Applied Mechanics (ISTAM 2019).

[127] Siao Y., Wen C. (2021). Examination of Molten Pool with Marangoni Flow and Evaporation Effect by Simulation and Experiment in Selective Laser Melting. Int. Commun. Heat Mass Transf..

[128] Lee K.H., Yun G.J. (2020). A Novel Heat Source Model for Analysis of Melt Pool Evolution in Selective Laser Melting Process. Addit. Manuf..

[129] Mukherjee T., Wei H.L., De A., DebRoy T. (2018). Heat and Fluid Flow in Additive Manufacturing—Part II: Powder Bed Fusion of Stainless Steel, and Titanium, Nickel and Aluminum Base Alloys. Comput. Mater. Sci..

[130] Körner C., Bauereiß A., Attar E. (2013). Fundamental Consolidation Mechanisms during Selective Beam Melting of Powders. Model. Simul. Mater. Sci. Eng..

[131] Korzekwa D.A. (2009). Truchas—A Multi-Physics Tool for Casting Simulation. Int. J. Cast Met. Res..

[132] Furumoto T., Ueda T., Abdul Aziz M.S., Hosokawa A., Tanaka R. (2010). Study on Reduction of Residual Stress Induced during Rapid Tooling Process: Influence of Heating Conditions on Residual Stress. Key Eng. Mater..

[133] Zhong Y., Rännar L., Wikman S., Koptyug A., Liu L., Cui D., Shen Z. (2017). Additive Manufacturing of ITER First Wall Panel Parts by Two Approaches: Selective Laser Melting and Electron Beam Melting. Fusion Eng. Des..

[134] Yakout M., Elbestawi M.A., Veldhuis S.C. (2019). Density and Mechanical Properties in Selective Laser Melting of Invar 36 and Stainless Steel 316L. J. Mater. Process. Technol..

[135] Yakout M., Elbestawi M.A., Veldhuis S.C., Nangle-Smith S. (2020). Influence of Thermal Properties on Residual Stresses in SLM of Aerospace Alloys. Rapid Prototyp. J..

[136] Gusarov A.V., Pavlov M., Smurov I. (2011). Residual Stresses at Laser Surface Remelting and Additive Manufacturing. Phys. Procedia.

[137] Keller N., Ploshikhin V. New Method for Fast Predictions of Residual Stress and Distortion of AM Parts. Proceedings of the 2014 International Solid Freeform Fabrication Symposium—An Additional Manufacturing Conference SFF 2014.

[138] Alvarez P., Ecenarro J., Setien I., Sebastian M.S., Echeverria A., Eciolaza L. (2016). Computationally Efficient Distortion Prediction in Powder Bed Fusion Additive Manufacturing. Int. J. Eng. Res. Sci..

[139] Yadroitsev I., Krakhmalev P., Yadroitsava I. (2014). Selective Laser Melting of Ti6Al4V Alloy for Biomedical Applications: Temperature Monitoring and Microstructural Evolution. J. Alloys Compd..

[140] Jiang W., Dalgarno K.W., Childs T.H.C. Finite Element Analysis of Residual Stresses and Deformations in Direct Metal SLS Process. Proceedings of the Solid Freeform Fabrication 2002 Symposium.

[141] Ansari M.J., Nguyen D., Park H.S. (2019). Investigation of SLM Process in Terms of Temperature Distribution and Melting Pool Size: Modeling and Experimental Approaches. Materials.

[142] Kong F., Kovacevic R. (2010). 3D Finite Element Modeling of the Thermally Induced Residual Stress in the Hybrid Laser/Arc Welding of Lap Joint. J. Mater. Process. Technol..

[143] Zaeh M.F., Branner G. (2010). Investigations on Residual Stresses and Deformations in Selective Laser Melting. Prod. Eng..

[144] Paul R., Anand S. (2011). Optimal Part Orientation in Rapid Manufacturing Process for Achieving Geometric Tolerances. J. Manuf. Syst..

[145] Phatak A.M., Pande S.S. (2012). Optimum Part Orientation in Rapid Prototyping Using Genetic Algorithm. J. Manuf. Syst..

[146] Aziz M.S.A., Furumoto T., Ueda T., Abe S., Hosokawa A., Tanaka R. (2012). Study on Thermal and Strain Behaviour in Selective Laser Sintering Process. Key Eng. Mater..

[147] Bayat M., Dong W., Thorborg J., To A.C., Hattel J.H. (2021). A Review of Multi-Scale and Multi-Physics Simulations of Metal Additive Manufacturing Processes with Focus on Modeling Strategies. Addit. Manuf..

[148] Khairallah S.A., Anderson A.T., Rubenchik A., King W.E. (2016). Laser Powder-Bed Fusion Additive Manufacturing: Physics of Complex Melt Flow and Formation Mechanisms of Pores, Spatter, and Denudation Zones. Acta Mater..

[149] Rodgers T.M., Madison J.D., Tikare V. (2017). Simulation of Metal Additive Manufacturing Microstructures Using Kinetic Monte Carlo. Comput. Mater. Sci..

[150] Sreeramagiri P., Bhagavatam A., Alrehaili H., Dinda G. (2020). Direct Laser Metal Deposition of René 108 Single Crystal Superalloy. J. Alloys Compd..

[151] Carter L.N., Martin C., Withers P.J., Attallah M.M. (2014). The Influence of the Laser Scan Strategy on Grain Structure and Cracking Behaviour in SLM Powder-Bed Fabricated Nickel Superalloy. J. Alloys Compd..

[152] Pauza J.G., Tayon W.A., Rollett A.D. (2021). Computer Simulation of Microstructure Development in Powder-Bed Additive Manufacturing with Crystallographic Texture. Model. Simul. Mater. Sci. Eng..

[153] Liu P., Wang Z., Xiao Y., Horstemeyer M.F., Cui X., Chen L. (2019). Insight into the Mechanisms of Columnar to Equiaxed Grain Transition during Metallic Additive Manufacturing. Addit. Manuf..

[154] Yan W., Ge W., Qian Y., Lin S., Zhou B., Liu W.K., Lin F., Wagner G.J. (2017). Multi-Physics Modeling of Single/Multiple-Track Defect Mechanisms in Electron Beam Selective Melting. Acta Mater..

[155] Sreeramagiri P., Bhagavatam A., Ramakrishnan A., Alrehaili H., Dinda G.P. (2020). Design and Development of a High-Performance Ni-Based Superalloy WSU 150 for Additive Manufacturing. J. Mater. Sci. Technol..

[156] Mishra R.S., Thapliyal S. (2021). Design Approaches for Printability-Performance Synergy in Al Alloys for Laser-Powder Bed Additive Manufacturing. Mater. Des..

[157] Irwin J., Reutzel E.W., Michaleris P., Keist J., Nassar A.R. (2016). Predicting Microstructure From Thermal History During Additive Manufacturing for Ti-6Al-4V. J. Manuf. Sci. Eng..

[158] Rodgers T.M., Mitchell J.A., Tikare V. (2017). A Monte Carlo Model for 3D Grain Evolution during Welding. Model. Simul. Mater. Sci. Eng..

[159] Gandin C.A., Desbiolles J.L., Rappaz M., Thevoz P. (1999). A Three-Dimensional Cellular Automation-Finite Element Model for the Prediction of Solidification Grain Structures. Metall. Mater. Trans. A.

[160] Ai Y., Jiang P., Wang C., Mi G., Geng S. (2018). Experimental and Numerical Analysis of Molten Pool and Keyhole Profile during High-Power Deep-Penetration Laser Welding. Int. J. Heat Mass Transf..

[161] Chen H., Fang Q., Zhou K., Liu Y., Li J. (2020). Unraveling Atomic-Scale Crystallization and Microstructural Evolution of a Selective Laser Melted FeCrNi Medium-Entropy Alloy. CrystEngComm.

[162] Waqar S., Sun Q., Liu J., Guo K., Sun J. (2021). Numerical Investigation of Thermal Behavior and Melt Pool Morphology in Multi-Track Multi-Layer Selective Laser Melting of the 316L Steel. Int. J. Adv. Manuf. Technol..

[163] Harrison N.J., Todd I., Mumtaz K. (2015). Reduction of Micro-Cracking in Nickel Superalloys Processed by Selective Laser Melting: A Fundamental Alloy Design Approach. Acta Mater..

[164] Nie P., Ojo O.A., Li Z. (2014). Numerical Modeling of Microstructure Evolution during Laser Additive Manufacturing of a Nickel-Based Superalloy. Acta Mater..

[165] Zhang Z., Demir K.G., Gu G.X. (2019). Developments in 4D-Printing: A Review on Current Smart Materials, Technologies, and Applications. Int. J. Smart Nano Mater..

[166] Skylar-Scott M.A., Mueller J., Visser C.W., Lewis J.A. (2019). Voxelated Soft Matter via Multimaterial Multinozzle 3D Printing. Nature.

[167] Vangelatos Z., Zhang Z., Gu G.X., Grigoropoulos C.P. (2020). Tailoring the Dynamic Actuation of 3D-Printed Mechanical Metamaterials through Inherent and Extrinsic Instabilities. Adv. Eng. Mater..

[168] Jin Z., Zhang Z., Demir K., Gu G.X. (2020). Machine Learning for Advanced Additive Manufacturing. Matter.

[169] Heeling T., Cloots M., Wegener K. (2017). Melt Pool Simulation for the Evaluation of Process Parameters in Selective Laser Melting. Addit. Manuf..

[170] Gardner J.M., Hunt K.A., Ebel A.B., Rose E.S., Zylich S.C., Jensen B.D., Wise K.E., Siochi E.J., Sauti G. (2019). Machines as Craftsmen: Localized Parameter Setting Optimization for Fused Filament Fabrication 3D Printing. Adv. Mater. Technol..

[171] Jin Z., Zhang Z., Gu G.X. (2019). Autonomous In-Situ Correction of Fused Deposition Modeling Printers Using Computer Vision and Deep Learning. Manuf. Lett..

[172] Gobert C., Reutzel E.W., Petrich J., Nassar A.R., Phoha S. (2018). Application of Supervised Machine Learning for Defect Detection during Metallic Powder Bed Fusion Additive Manufacturing Using High Resolution Imaging. Addit. Manuf..

[173] Zhu Z., Anwer N., Huang Q., Mathieu L. (2018). Machine Learning in Tolerancing for Additive Manufacturing. CIRP Ann..

[174] Mozaffar M., Paul A., Al-Bahrani R., Wolff S., Choudhary A., Agrawal A., Ehmann K., Cao J. (2018). Data-Driven Prediction of the High-Dimensional Thermal History in Directed Energy Deposition Processes via Recurrent Neural Networks. Manuf. Lett..

[175] Khanzadeh M., Rao P., Jafari-Marandi R., Smith B.K., Tschopp M.A., Bian L. (2018). Quantifying Geometric Accuracy with Unsupervised Machine Learning: Using Self-Organizing Map on Fused Filament Fabrication Additive Manufacturing Parts. J. Manuf. Sci. Eng. Trans. ASME.

[176] Yi H., Park E., Kim S. (2018). Multi-Agent Deep Reinforcement Learning for Autonomous Driving. KIISE Trans. Comput. Pract..

[177] Garaigordobil A., Ansola R., Santamaría J., Fernández de Bustos I. (2018). A New Overhang Constraint for Topology Optimization of Self-Supporting Structures in Additive Manufacturing. Struct. Multidiscip. Optim..

[178] Zhang W., Zhou L. (2018). Topology Optimization of Self-Supporting Structures with Polygon Features for Additive Manufacturing. Comput. Methods Appl. Mech. Eng..

[179] Hiller J., Lipson H. (2009). Design and Analysis of Digital Materials for Physical 3D Voxel Printing. Rapid Prototyp. J..

[180] Kappes B., Moorthy S., Drake D., Geerlings H., Stebner A., Ott E., Liu X., Andersson J., Bi Z., Bockenstedt K., Dempster I., Groh J., Heck K., Jablonski P., Kaplan M. (2018). Proceedings of the 9th International Symposium on Superalloy 718 & Derivatives: Energy, Aerospace, and Industrial Applications, Pittsburgh, PA, USA, 14–18 September 2018.

[181] Holzmond O., Li X. (2017). In Situ Real Time Defect Detection of 3D Printed Parts. Addit. Manuf..

[182] Yuanbin W., Pai Z., Xun X., Huayong Y., Jun Z. (2019). Production Planning for Cloud-Based Additive Manufacturing—A Computer Vision-Based Approach. Robot. Comput. Integr. Manuf..

[183] Scime L., Beuth J. (2018). Anomaly Detection and Classification in a Laser Powder Bed Additive Manufacturing Process Using a Trained Computer Vision Algorithm. Addit. Manuf..

[184] Nascimento R., Martins I., Dutra T.A., Moreira L. (2023). Computer Vision Based Quality Control for Additive Manufacturing Parts. Int. J. Adv. Manuf. Technol..

[185] Zhang J., Habibnejad-korayem M., Liu Z., Lyu T., Sun Q., Zou Y. (2021). A Computer Vision Approach to Evaluate Powder Flowability for Metal Additive Manufacturing. Integr. Mater. Manuf. Innov..

[186] Ward L., Agrawal A., Choudhary A., Wolverton C. (2016). A General-Purpose Machine Learning Framework for Predicting Properties of Inorganic Materials. npj Comput. Mater..

[187] Gu G.X., Chen C.T., Richmond D.J., Buehler M.J. (2018). Bioinspired Hierarchical Composite Design Using Machine Learning: Simulation, Additive Manufacturing, and Experiment. Mater. Horiz..

[188] Braconnier D.J., Jensen R.E., Peterson A.M. (2020). Processing Parameter Correlations in Material Extrusion Additive Manufacturing. Addit. Manuf..

[189] Jin Z., Zhang Z., Gu G.X. (2020). Automated Real-Time Detection and Prediction of Interlayer Imperfections in Additive Manufacturing Processes Using Artificial Intelligence. Adv. Intell. Syst..

